# Progress in Modern Marine Biomaterials Research

**DOI:** 10.3390/md18120589

**Published:** 2020-11-25

**Authors:** Yuliya Khrunyk, Slawomir Lach, Iaroslav Petrenko, Hermann Ehrlich

**Affiliations:** 1 Department of Heat Treatment and Physics of Metal, Ural Federal University, 620002 Ekaterinburg, Russia; iu.ia.khrunyk@urfu.ru; 2 Institute of High Temperature Electrochemistry, Ural Branch, Russian Academy of Sciences, 620990 Ekaterinburg, Russia; 3 Department of Biomedical Chemistry, Faculty of Chemistry, University of Gdansk, 80-308 Gdansk, Poland; slawomir.lach@ug.edu.pl; 4 Institute of Electronics and Sensor Materials, Technische Universität Bergakademie Freiberg, 09599 Freiberg, Germany; Iaroslav.petrenko@esm.tu-freiberg.de; 5 Center for Advanced Technology, Adam Mickiewicz University, 61614 Poznan, Poland

**Keywords:** marine biomaterials, algal polysaccharides, chitin, spongin, collagen, gelatin, keratin, conchiolin, corals, biominerals

## Abstract

The growing demand for new, sophisticated, multifunctional materials has brought natural structural composites into focus, since they underwent a substantial optimization during long evolutionary selection pressure and adaptation processes. Marine biological materials are the most important sources of both inspiration for biomimetics and of raw materials for practical applications in technology and biomedicine. The use of marine natural products as multifunctional biomaterials is currently undergoing a renaissance in the modern materials science. The diversity of marine biomaterials, their forms and fields of application are highlighted in this review. We will discuss the challenges, solutions, and future directions of modern marine biomaterialogy using a thorough analysis of scientific sources over the past ten years.

## 1. Introduction

**Figure 1:**
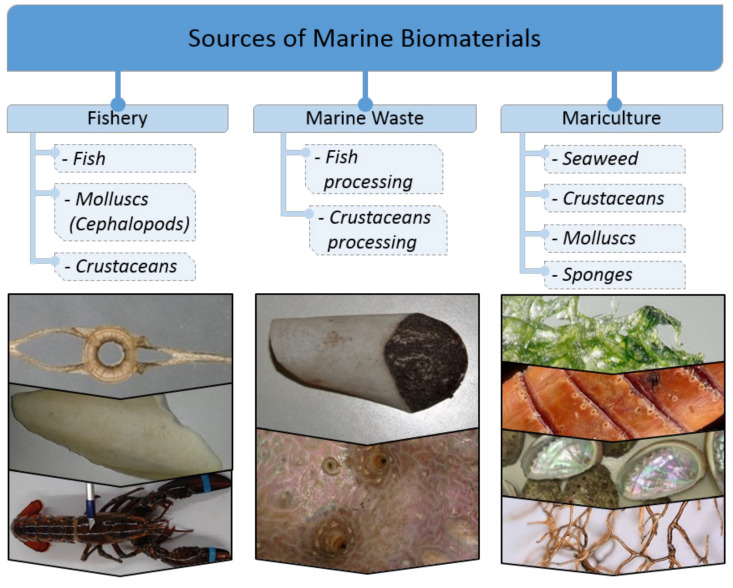
Overview of the main sources of marine biomaterials used nowadays.

Humanity has been using marine biomaterials since ancient times (i.e., molluscan shells, corals, bath sponges skeletons, byssus threads), but reaching an industrial level today has become real thanks to the rapid development of various kinds of processing technologies and maricultures. Often, there is a possibility of the utilization of fish, molluscs, or marine arthropod processing products in order to use them most efficiently and not only for feed production. Due to the absence of possible human pathogens in marine biomaterials, a number of them (i.e., collagen, gelatin, keratin) have become an alternative source of long and well-established biopolymers in medicine and cosmetics. Today, modern scaffolding strategies [1,2,3] for tissue engineering are based on the application of diverse already naturally pre-fabricated 3D skeletal constructs of marine invertebrates origin [4]. Sources of marine biomaterials are still plentiful [5] in spite of partial overfishing, dramatic climate changes, and the increasing pollution of the world’s oceans with industrial waste. An attempt to classify marine biomaterials by their origin is presented by us in Figure 1.

Due to the huge amount of scientific information available in various scientific sources, we considered carrying out its analysis to be expedient, choosing certain topics that include marine polysaccharides of invertebrates and algal origin, marine structural proteins (spongin, collagen, gelatin, keratin, conchiolin) as well as marine biominerals from corals and molluscan shells. For the first time, in order to facilitate the perception of large volumes of information and focus on especially important parameters characterizing a particular biomaterial, we took the liberty of presenting a part of information in the form of so-called “Biomaterial passports” (see Table 1, Table 2, Table 3, Table 4, Table 5, Table 6, Table 7, Table 8, Table 9, Table 10, Table 11, Table 12 and Table 13). This form includes scientific name, chemical formula, molecular weight, physicochemical and material properties, extraction methods, market and patent situation of corresponding biological materials discussed in this article. For brevity, some aspects will only be briefly discussed, but interested readers are referred to pertinent references.

This review has the ambitious goal to provide a thorough and comprehensive coverage of marine biomaterials as multifaceted topic. Consequently, we strongly believe that numerous open questions raised in this review will inspire a younger generation of experts in marine biology, biochemistry, bioengineering, biomimetics, bioinspired materials science, biomineralization, marine waste processing, fishery and mariculture to research marine biomaterials as examples of renewable natural sources which stood the test of time through evolutionary development of corresponding organisms.

## 2. Marine Polysaccharides

Polysaccharides belong to biological materials with carbohydrate backbone-based structures. In this review, we focus attention only on structural aminopolysaccharide chitin and selected polysaccharides of algal origin. Chitosan, an artificially produced derivate of chitin, was not the goal of our analytical research exclusively due to the existence of numerous reviews related to this biopolymer (i.e., [6,7,8,9,10,11]).

### 2.1. Chitin

The main characteristics of chitin are summarized in Table 1.

**Table 1:** Biomaterial passport: chitin.

Scientific Name	Chitin
Chemical formula, MW, chemical structure, polymorphism	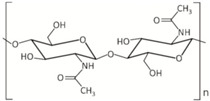
	(C_8_H_13_O_5_N)*_n_*; MW ranges from several to thousands of kDa [12]. Chitin is a linear polymer of *N*-acetyl-d-glucosamine units that are linked by 1,4-β-glycosidic bonds [13]. It exists in three crystalline polymorphic forms: α-, β-and γ-chitin [14,15]. Marine sources of α-chitin: crustaceans, sponges; of β-chitin: cephalopods [16].
Physicochemical properties	Due to its semicrystalline structure and hydrophobicity chitin is not soluble in usual solvents, i.e., water, the most organic solvents, though it shows solubility in hexafluoroacetone sesquihydrate, hexafluoroisopropanol, chloroalcohols (with sulfuric acid), mixture of dimethylacetamide with 5% lithium chloride [17] and diverse ionic liqiuds [18].
Chitin extraction/Physical form after extraction	For commercial purposes, chitin is extracted using chemical, electrochemical and biochemical methods from the cuticles of crustaceans, mostly crabs and shripms [19,20,21,22,23,24] and corals [25]. It is isolated by chemical extraction via three stages, i.e., deproteinization by alkaline treatment, i.e., employing NaOH, Na_2_CO_3_, NaHCO_3_, KOH, K_2_CO_3_, demineralization using acidic (i.e., HCl, HNO_3_, H_2_SO_4_, CH_3_COOH), or EDTA-based solutions [26], and finally discoloration following the incubation in alkaline solution or by the addition of acetone or, alternatively, using KMnO_4_, H_2_O_2_ [27] or oxalic acid [12,28]. Currently, numerous studies aimed at developing different protocols to isolate chitin from seafood shells [29,30,31,32,33,34] as well as marine sponges [35] have been reported. Chitin is extracted in the form of flakes, powders, and scaffolds.
Biomaterials properties (biocompatibility, biodegradability, toxicity, immune responses)	Elastic (Young’s) modulus ranges from 92 GPa [36] to 4 GPa [37]. Thermostability: 260–360 °C [38,39,40]. Biocompatible [1,41,42,43] and biodegradable [12]; can be hydrolyzed by chitinases [44]; non-toxic and [45] of low immunogenicity [46,47].
Market situation (world market reports)	According to Global Industry Analysts, Inc. data, global chitin and chitosan market was predicted to reach US $4.2 billion by 2021 [12].
Patents	Currently, about several hundreds of patents on the extraction and modification of chitin and its derivatives as well as their applications exist.
	For search, use: https://patents.google.com/
	Selected examples:
	US6310188B1. Method for producing chitin or chitosan
	US6632941B2. Method of extracting chitin from the shells of exoskeletal animals
	CN106496362A. The extracting method of chitin in a kind of Carapax *Eriocheir sinensis*
	US20180186899A1. Compositions of partially deacetylated chitin derivatives
	JP2822174B2. Method for producing chitin chitosan fiber and structure
	US5623064A. Poly-β-1→-4-N-acetylglucosamine
	US9433698B2. High strength chitin composite material and method of making
	US9708634B2. Process for making chitin derivatives
	US7241463B2. Methods for processing crustacean material
	US4066735A. Process for demineralization of crustacea shells
	US4293098A. Recovery of active chitin and enhanced protein meal
	WO1986006082A1. A process for recovering chitin from materials in which chitin occurs together with or connected to proteinaceous substances
	US5053113A. Method of chitin production from chitin containing raw materials
	JPH05310804A. Production of chitin or chitosan from integument of crustacea

### 2.2. Recent Studies in Crustacean Chitin Applications

Crustaceans (lobster, crab and krill) chitin [48,49] including chitin-based cuticles of more than 300 million tons of Antarctic krill present in the world ocean [50], remains the main industrial source of this structural biopolymer.

Importantly, crustacean shells combined with commercial chitin can be used as biosorbents to remove heavy metals from surface runoff that solves two environmental problems: the use of seafood wastes and water resources management [51]. Moreover, seafood wastes can be employed in agriculture: the use of shrimp chitin as feed additives showed a positive effect on growth and carcass characteristics of broiler chickens [52]. Another application of crustacean shells was shown in a recent research of Las Heras et al. [53] who described the generation of chitin-containing sponge like scaffolds, which were biocompatible with human mesenchymal stromal cells (hMSCs), thus representing a high potential for biomedical technologies, in particular, for tissue engineering. Likewise, novel interesting scaffolds-candidates for tissue engineering were designed from crab shells chitin and silk protein fibroin obtained from silkworm *Antheraea pernyi* cocoons [41]. Finally, nanomaterials from shrimp chitin (nanocrystals and nanofibers) were reported to have no cytotoxic effect, which was tested with epithelial-like and fibroblast-like cell lines [54]: this research indicates that such components can be safely used in biomedical industry.

### 2.3. Poriferan Chitin: Progress in the APPLICATion of Poriferan Chitinous Scaffolds

The presence of chitin in marine sponges has been revealed only recently [55,56] that was further confirmed by the detection of chitin in fossilized skeleton of 505 MYR old demosponge *Vauxia gracilents* [39]. Since then, chitin has been isolated from numerous species of marine [25,43,55,56,57,58,59,60,61,62,63,64,65,66,67,68,69] as well as fresh-water sponges [70].

Over the last decade, 3D chitinous scaffolds of poriferan origin were reported to have a huge potential for biomedical applications due to ability of corresponding sponges to grow under marine farming conditions [71]. Indeed, being biocompatible and supporting cell adhesion, growth, and proliferation, these scaffolds serve as perfect ready-to-use 3D matrices for tissue engineering and regenerative medicine [3,4,43,72,73,74]. For example, hMSCs seeded onto *Aplysina aerophoba* [61], *A. fulva* [1], and *Ianthella basta* [62] chitinous scaffolds displayed good attachment, viability, proliferation, and the capability of differentiation into osteogenic (*A. aerophoba, A. fulva*), adipogenic (*A. aerophoba, I. basta*) and chondrogenic (*A. aerophoba*) lineages, provided that the growth media were supplemented with respective differentiation inducers. Furthermore, chitinous scaffolds from *I. labyrinthus* were applied for the cultivation of human induced pluripotent stem cell-derived cardiomyocytes (iPSC-CMs): the long-term study (20 days) demonstrated that the cells grown on investigated scaffolds formed contracting cell clusters indicating that *I. labyrinthus* chitin is a source of suitable matrices to conduct research on the regeneration of myocardial tissue [66]. Additionally, poriferan chitinous scaffolds can serve as templates for co-culture systems mimicking in vivo processes [1,6]. A recent study conducted by our group [75,76] explored the ability of hemocytes from *Cornu aspersum* snail to grow on chitinous scaffold of *A. archeri*, which resulted in the generation of a new calcium-layered biomimetic product.

**Figure 2:**
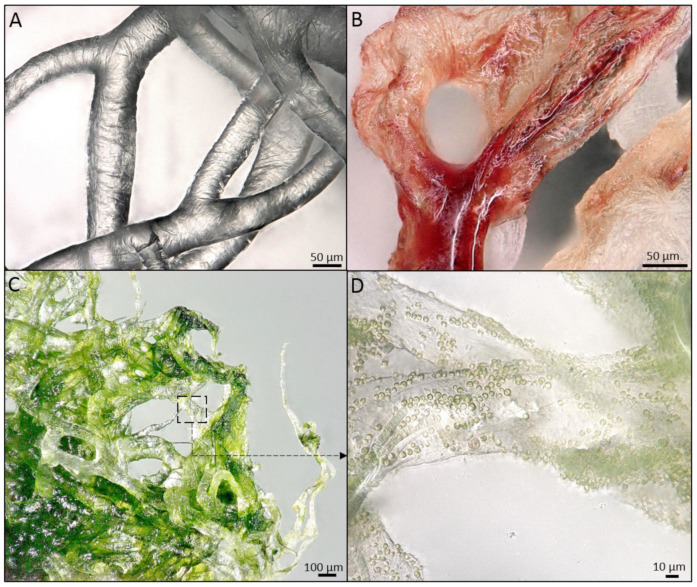
Digital microscopy images: Naturally prefabricated 3D chitinous skeletal constructs of verongiids sponges origin (**A**) are made of interconnected tube-like fibres with excellent ability to absorb diverse liquids including blood (**B**). They can be used also as biodegradable 3D scaffold-based bioreactors for cultivation of algal cultures (**C**,**D**) for further production of corresponding biologically active compounds under controlled laboratory conditions.

In addition, the study of sponge chitinous scaffolds, i.e., scaffolds of *Ianthella* species, pointed to their elasticity and capillary effect that allows these unique matrices to assume the shape of the objects they were placed on and swell with liquids (i.e., blood), the properties, which can be used in wound treatment [35,66] (Figure 2). In addition, owing to their capillary effect, sponge chitinous scaffolds can be used as adsorbents of crude oil and synthetic dyes [35] as well as drug delivery systems, which was shown for a sponge scaffold adsorbing antimicrobial drug decamethoxine leading to the inhibition of *Staphylococcus aureus* pathogene [68]. Another important application of sponge chitinous scaffolds lies in waste water treatment, as was shown for the case of *A. aerophoba* adsorbing uranium [77].

Intriguingly, sponge chitinous scaffolds serve as a source of inspiration for biomimetic research including the development of diverse composite materials using “*extreme biomimetics route*” [2,78,79]. Some sponges of Verongiida order were reported to carry unique biocomposites composed of amorphous silica, crystalline aragonite and chitin [74] that can be used as a «natural example» of chitin mineralization. Recently, a great progress in the application of sponge chitinous scaffolds as matrices for metal incorporation has been reported. For example, isolated chitinous skeleton of *A. fulva* demosponge was shown to undergo electrochemical deposition of copper following “sensibilization” employing silver nitrate solution [80,81]. Other examples include *A. aerophoba* scaffolds covered by ZrO_2_ [82] and Fe_2_O_3_ [83], the use of *A. cauliformis* as a template for the growth of GeO_2_ nanocrystals [84], and nanosilica depositions onto *I. basta* scaffolds [85]. All such treatments were conducted under hydrothermal conditions up to 185 °C. Such composites could be used for waste water treatment and photocatalytic decomposition of water to convert solar energy into chemical energy [81,86]. These findings are of a very high relevance to the current development in EV industry including the production of sensors, catalysts, electrochemical capacitors, the research focused on the latter also included I. basta chitinous scaffold to produce chitosan/sponge chitin membrane [87]. Finally, poriferan chitin-containing biocomposite materials demonstrated a potential for the adsorption of water pollutants, i.e., *A. archeri* scaffolds were used as a matrix for the immobilization of *Trametes versicolor* laccase that proved to efficiently remove tetracycline [69], while metallization of *A. aerophoba* scaffolds with silver nanoparticles and AgBr proved to be promising for water filtering systems with antibacterial properties [88].

Modern biocomposite-based scaffolding strategies include two key ways: to produce requested 3D constructs from corresponding precursors using technological tools or simply use naturally already pre-fabricated scaffolds if they originate from renewable sources. Such kind of 3D scaffolds remains to be one of the crucial features of skeletons of marine sponges that belong to the Verongiida order inhabiting oceans since the Precambrian [39].

Fabrication of biomimetic materials and scaffolds is usually a micro- or even nanoscale process. However, mostly all practical applications on the industrial level require larger-scale synthesis of nanoscale features. Recent development in micro-CT tomography and 3D printing not only bring us closer to the biomimicry of hierarchical 3D open cell hierarchical structures, but also clearly shows how nature is ahead of our most advanced technologies. Nevertheless, science still can benefit from the remarkable structural advancements of natural chitin-based scaffolds by simply applying them as multi-target templates in biomedicine and various modern technologies.

### 2.4. Polysaccharides of Algal Origin

Historically, the value of marine macroalgae (seaweeds) was greatly underestimated. Already in ancient Greece, Virgil and Horace, while referring to something completely worthless, used the term “villior alga” [89]. Seaweeds can be divided into green, red and brown algae containing a variety of polysaccharides [90,91], the properties of which were extensively studied during the last decade. Almost all representatives of brown algae are marine, mostly occurring in cold water, especially in the northern latitudes [92], and are rich in polysaccharides such as alginates (Table 2) and fucoidans (Table 3).

**Table 2:** Biomaterial passport: alginates.

Scientific Name	Alginates
Chemical structure, MW	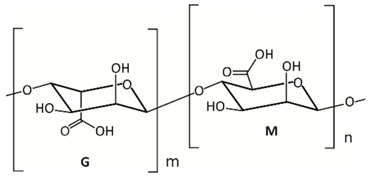
	Alginates are salts of alginic acid, a linear polymer composed of blocks of β-d mannuronic acid (M) and α-l guluronic acid (G) residues linked by 1-4 glycosidic bonds [90,93]. The molecular weight of alginic acid and its salts ranges from 5 to 20 kDa [94].
Physicochemical properties	Phycocolloids are known to form viscous solutions or gels [95]. Over 200 alginates with different physicochemical properties are produced [96]. Alginates can efficiently bind divalent cations, which results in hydrogel formation and crosslinked polymeric scaffolds [97]. The presence of *O*-acetyl groups, which was shown for algal alginates [98], increases polymer solubility affecting physicochemical parameters such as viscoelasticity and molecular mass [99].
Alginate extraction/Physical form after extraction	Alginates are produced industrially from marine seaweeds, which belong to brown algae [90]. Conventional extraction of alginates consists of the following five steps: (i) acidification of seaweeds; (ii) alkaline extraction using Na_2_CO_3_; (iii) solid/liquid separation; (iv) precipitation and (v) drying [100]. In addition, seaweed tissue can be softened and bleached using formaldehyde/formalin [100]. About 25% of alginate yield can be achieved in 2 h; however, the extraction can be conducted much faster (in 15–30 min) using ultrasound treatment [101]. Usually, alginates are extracted as dry, powdered sodium alginate [95]. Alginates and their derivatives are widely used as stabilizers, thickeners, viscosifiers, additives, gel and film formers [99].
Biomaterials properties (biocompatibility, biodegradability, toxicity, immune responses)	In algae, being constituents of cell wall and inter-cellular matrix, alginates provide mechanical strength and flexibility necessary for the survival in water [100]. Due to their non-toxicity, biocompatibility, biodegradability, non-immunogenicity, and hydrophilicity alginates have a great potential for pharmaceutical and biomedical applications [99].
Market situation (world market reports)	Owing to their properties such as thickeners, the ability to form gels, sodium, and calcium films alginates are widely applied in the food, printing, dyeing, textile, pharmaceutical, and cosmetic industries. According to the report of Market Data Forecast [102], the global alginates market was estimated as USD 409.2 million in 2020 and is expected to reach USD 529.2 million by 2025. Alginates market is predicted to grow mainly in Europe and Asia Pacific.
Patents	Currently, about several hundreds of patents on the extraction and modification of alginic acid and its derivatives as well as their applications exist.
	For search, use: https://patents.google.com/
	Selected examples:
	US2653106A. Manufacture of alginates
	US20150289533A1. Alginate gum
	US8741872B2. Self-gelling alginate systems and uses thereof
	US2420308A. Gel-forming algin composition and method
	US1814981A. Process of preparing alginic acid and compounds thereof
	EP0345886A2. Alginate gels
	US5266326A. In situ modification of alginate
	EP0849281A1. Bioresorbable alginate derivatives
	US5874100A. Alginate fibres, manufacture and use
	WO2000009566A1. Method for producing ultra-pure alginates
	US6150581A. Chitosan/alginate anti-adhesion barrier
	US6432449B1. Biodegradable sustained-release alginate gels
	US10292936B2. Modified alginates for cell encapsulation and cell therapy
	US10426735B2. Modified alginates for anti-fibrotic materials and applications

As represented below, alginates as biomaterials are widely applied in diverse biomedical fields. Being used as polymeric coating for therapeutic agents, so-called alginate microspheres can be applied for the delivery of different drugs [97,103,104,105,106,107,108,109,110] including tetracycline derivative minocycline [111] and vancomycin [112] antibiotics, lipopolysaccharide subunit antigen as vaccination therapy against *Klebsiella pneumoniae* [113], paracetamol [114], and anticancer drugs [115]. Additionally, alginates, in the form of hydrogels or composites, in particular, employing bioprinting [116,117,118,119,120,121], are widely employed in tissue engineering, such as tissue engineering of bone [122,123,124,125,126,127,128,129], cartilage [130,131], skin [132,133], muscle [134], and even neural tissue engineering [135] as well as cardiac regeneration [136]. Recently, alginates were reported to be widely researched for wound healing applications [137,138,139,140,141,142,143].

**Table 3:** Biomaterial passport: fucoidans.

Scientific Name	Fucoidans
Chemical Structure, MW	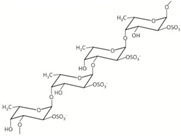
	Fucoidans are sulphated hetero-polysaccharides consisting of α 1-3 linked sulphated L fucose with repeating sequence of alternating α 1-3 and α 1-4 glycosidic bonds [90,144]. MW of most fucoidans was reported to vary within 200–2000 kDa [145].
Physicochemical properties	Isolated shielded opposite groups contribute to the solubility of fucoidans in solvents with higher dielectric constants, such as water, whereas solvents of lower dielectric constants, i.e., ethanol can be used for precipitation and isolation of fucoidans from other co-extracted natural compounds [146]. Fucoidan molecules, being stable in salts, i.e., NaCl and CaCl_2_, acid and alkaline solutions, are suitable for the use as stabilizing, thickening, and water-holding agents [147].
Fucoidan extraction/Physical form after extraction	Fucoidans can be extracted from brown algae such as *Undaria pinnatifida* (Miyeok), a common Korean edible brown seaweed [148], by hot acidic, alkaline, enzyme-, microwave- and ultrasound-assisted aqueous methods [146]. They are extracted in the form of fluffy, hygroscopic powders, soluble in water, relatively soluble in dimethyl sulfoxide (DMSO), but insoluble in ethanol [146].
Biomaterials properties (biocompatibility, biodegradability, toxicity, immune responses)	Fucoidans have specific mechanical properties. Indeed, these polysaccharides provide mechanical stability to brown seaweeds, in particular, they prevent the desiccation of the thallus tissues, especially at the lower tide levels or high summer temperatures [149]. Fucoidans were reported to be biocompatible, biodegradable and demonstrated low cytotoxicity and immunogenicity [144,150,151,152]. Some studies, however, pointed to their cytotoxicity in vitro and in vivo, which paves the way to their use as anticancer agents [153].
Market situation (world market reports)	Based on the New Research Analysis, the global fucoidan market size was USD 30 million in 2019 and is expected to reach USD 37 million in 2024 with Asia (mainly China and Japan) and the U.S.A. being the largest fucoidan consumption regions [154].
Patents	Currently, about several hundreds of patents on the extraction and applications of fucoidans exist.
	For search, use: https://patents.google.com/
	Selected examples:
	US20070087996A1. Method of extracting fucoidan
	US20100056473A1. Method of extracting fucoidan
	CN101993501A. Method for preparing fucoidan
	CN103665179A. Extraction device for kelp fucoidan
	US20080089941A1. Fucoidan compositions and methods
	US20050129708A1. Fucoidan-based health food
	CA2253573C. Fucoidan-containing foods or beverages
	US20150328268A1. Marine Plants Extract for Wound Healing
	CN101954087B. Fucoidan medicinal carrier and preparation method thereof
	NZ610788A. Process for isolating fucoidan and laminarin from live, harvested seaweed

Similar to alginates, fucoidans proved to be valuable in tissue engineering [155,156,157,158,159], drug delivery [160,161,162,163], and wound healing [164,165,166].

Apart from brown seaweeds, read and green algae also produce unique polysaccharides. Brief information on carrageenans, extracted from red algae, is presented in Table 4.

**Table 4:** Biomaterial passport: carrageenans.

Scientific Name	Carrageenans
Chemical structure, MW	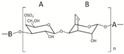
	Carrageenans, hydrophilic linear sulphated galactans, being composed of alternate units of d-galactose and 3,6-anhydro-galactose linked by α 1-3 and β 1-4 glycosidic bonds, are divided into groups, i.e., kappa (κ), iota (ι), lambda (λ), mu (μ), nu (υ), theta (θ) and others, which is based on their solubility in potassium chloride [90]. Their MW was reported to be within 200–800 kDa [167].
Physicochemical properties	Hydrocolloids of different solubility: κ-carrageenan is insoluble in cold water [168]; a higher hydrophilicity was shown for ι-carrageenan, while λ-carrageenan is freely soluble in water under most conditions [168] and even in cold milk [169]. λ-carrageenan is non-gelling and is used rather for its thickening properties and the ability to form creamy texture [169]. κ- and ι-carrageenans form gels [169] following heating [167,170] and cooling in the presence of K^+^, Ca^2+^, NH_4_^+^ cations [168]. ι-carrageenan is used to obtain soft gels [169] while κ-carrageenan, the main carrageenan applied in industry, forms strong, brittle gels the strength of which can be improved by locust bean gum, corn starch and wheat starch [168]. Thus, due to their specific texture properties carrageenans are widely used in food industry to improve appearance (creaminess, homogeneity), organoleptic qualities (juiciness, mouthfeel), and application (spreadability) [169].
Carrageenan extraction/Physical form after extraction	Carrageenans are extracted from various red algae species [90,171,172,173] by hot alkaline treatment followed by ethanol precipitation [168] in the form of translucent plates or powders [168,174].
Biomaterials properties (biocompatibility, biodegradability, toxicity, immune responses)	Carrageenans were shown to be biocompatible, biodegradable, non-immunogenic, and non-toxic compounds [19,175,176,177].
Market situation (world market reports)	The global carrageen market is predicted to reach USD 1.25 million by the end of 2024 [178].
Patents	Currently, about several hundreds of patents on the extraction and applications of carrageenans exist.
	For search, use: https://patents.google.com/
	Selected examples:
	US3956173A. Preparation of gels based on carrageenan
	US5502179A. Carrageenan product and a method of producing same
	US3094517A. Process for treating a polysaccharide of seaweeds of the gigartinaceae and solieriaceae families
	US3280102A. Preparation of carrageenan having improved water dispersibility
	US3907770A. Process of extracting carrageenan from seaweed
	JPS57202302A. Preparation of carrageenan
	US4443486A. Modified extractive of *Eucheuma cottonii* seaweed and composition containing same
	US6387354B1. Semi-refined carrageenan dentifrice binder
	WO2002048199A3. Production of carrageenan and carrageenan products
	SU756683A1. Method of obtaining jellifier from red algae
	CN103788225A. Production method of modified carrageenan
	AU2003245252A1. Carrageenan based antimicrobial compositions

Having viscosity increasing, stabilizing and gelling properties, carrageenans are widely used for controlled drug release, pharmaceuticals, food and other industries [174,179]. In addition, being biodegradable, these polysaccharides can be applied as films for food packaging: in order to increase their mechanical properties, nano-sized fillers such as melanin nanoparticles are employed as reinforcing agents [180]. Finally, carrageenans exhibit anticoagulant [181,182], antithrombotic [183], anti-HIV [151], antiviral [184,185,186], anti-cancer [187], immunomodulatory [177,188,189], and antioxidant [150,177,190] activities.

Green seaweeds, on the other hand, are abundant in ulvans (see Table 5).

**Table 5:** Biomaterial passport: ulvans.

Scientific Name	Ulvans
Chemical Structure, MW	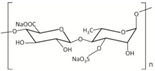
	Ulvans are composed of branched, complex structure without a defined backbone or a specific repeating monomer, usually consisting of rhamnose (17–45%), sulphate (14–23%), glucuronic acid (7–19%), xylose (2–12%), iduronic acid (1–9%), and glucose (1–7%) [191,192]. Their MW ranges from about 200 to 8200 kDa [193].
Physicochemical properties	Hydrocolloids, which in the presence of divalent cations, i.e., Ca^2+^, Cu^2+^, Zn^2+^, boric acid and slightly basic pH form gels [194]. At low and neutral pH, owing to rhamnose hydrophobicity, ulvans fold into beads-like conformation resulting in low viscosity [195,196]. At pH ~13, ulvans develop more open conformation leading to higher viscosities and gel strengths [196]. They have metal chelating ability, play the role of radical scavengers, and were shown to tolerate temperatures up to 180 °C [194].
Ulvans extraction/Physical form after extraction	Ulvans are extracted from green seaweeds [191,192]. Following cold water or hot water extraction and ethanol precipitation, they are recovered as fluffy powder [196].
Biomaterials properties (biocompatibility, biodegradability, toxicity, immune responses)	Ulvans were reported to be biocompatible, biodegradable, show a low toxicity, and immunogenicity [197,198].
Market situation (world market reports)	There is no open access data regarding a global ulvans market. It is known that ulvan containing green algae is consumed in Asian countries and are used in Chinese medicine [199]. Due to their high vitamin and fiber content, ulvans are also consumed in Europe [199]. The main ulvan producers are represented by China and Indonesia, which account for 49% and 37% of the world production, respectively [200].
Patents	Currently, about several hundreds of patents on the extraction and applications of ulvans exist.
	For search, use: https://patents.google.com/
	Selected examples:
	US7820176B2. Ulvans as activators of plant defense and resistance reactions against biotic or abiotic stresses
	FR2868252B1. Use of ulvanes as elicitors of nitrogen absorption mechanisms and protein synthesis
	EP2582810B1. Ulvan lyase, the method for manufacturing same, and uses thereof
	CA2562942C. Use of ulvans as elicitors of mechanisms for nitrogen absorption and protein synthesis
	WO2007045795A1. Product resulting from the grafting of fatty chains to ulvans and use of a said product as a surfactant
	US5089481A. Polysaccharides and antiviral drugs containing the same as active ingredient
	US20080083160A1.Compositions of enriched seaweeds in land-based sea water ponds
	US20080226740A1. Marine algal extracts comprising marine algal polysaccharides of low degree polymerizaton, and the preparation processes and uses thereof
	CN1108310C. Algae polysaccharide and its preparation and use

Biocompatibility of ulvans, shown in in vitro cell culture assays, enables their use in wound treatment [191,201] and as substrates for cell cultivation [202]. Like other algal polysaccharides with gelling properties, ulvans can be employed for drug delivery [203,204]. Furthermore, ulvans are used for the synthesis of silver nanoparticles, the antimicrobial activity of which are essential for cosmetic and biomedical industries [205]. Iduronic acid, another rare sugar component of ulvans, is reported to have anti-thrombotic activities [206]. Finally, similar to alginates and carrageenans, ulvans are used to produce films as biodegradable material for food packaging, antioxidant, optical, thermal, and mechanical characteristics of which can be modified [207].

Selected marine algae, belonging to the phylum Rhodophyta (red algae), have been recognized as renewable sources of such polysaccharides as agar (agar-agar) (see Table 6), agarose and agaropectin. Numerous methods of agar extraction from such algae species as *Gelidium*, *Acanthopeltis*, *Ceramium*, *Gracilaria*, and *Gloiopeltis* have been reported [208].

**Table 6:** Biomaterial passport: agar.

Scientific Name	Agar
Chemical structure, MW	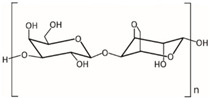
	Agars, (2*R*,3*S*,4*S*,5*R*)-2-(hydroxymethyl)-6-[[(4*R*,5*S*)-4-hydroxy-3-methyl-2,6-dioxabicyclo[3.2.1]octan-8-yl]oxy]-4-methoxyoxane-3,5-diol, are known as water-soluble, gel-forming polysaccharide extracts from agarophyte members of the Rhodophyta [209]. Agar is derived from the polysaccharide agarose, which forms the supporting structure in the cell walls of certain species of algae, and which is released on boiling. Average molecular weight of agar ranges between 35.7 and 144 kDa for commercial preparations [208].
Physicochemical properties	Insoluble in cold water. Main physical properties of agar include gel strength, gelling, and melting temperature [210,211].
Agar extraction/Physical form after extraction	Agar can be extracted with different yields from such algae as *Gelidium*, *Acanthopeltis*, *Ceramium*, *Gracilaria*, and *Gloiopeltis* species by boiling in 70, 60, 50% alcohol and water [208]. Two classical extraction methods of total agar extraction with and without NaOH treatment have been described as follows: “The dried sample of 30 g of algae was boiled for 2 h with 900 mL of distilled water and used for non-alkali treatment (native agar). Another 30 g sample was incubated in 2 L of 5% NaOH solution at 80 °C for 2 h. The algae were washed in running tap water for 30 min to remove excess NaOH. The alkali-treated algae were neutralized in 2% H_2_SO_4_ solution for 1 h, then washed in running tap water overnight until complete elimination of the acid” [209]. Agar scaffolds preparation for tissue engineering was also reported: “0.02% agar was soaked in distilled water for 30 min at room temperature and then boiled to 80 °C with stirring for 2 h until it completely turned into a transparent homogeneous solution. The agar solution was poured into a mold and cooled to room temperature” [212]. The development of agar-based bioaerogels [213] and membranes [214] has also been described.
Biomaterials properties (biocompatibility, biodegradability, toxicity, immune responses)	The gel-forming ability and solubility of agar polysaccharides rely on the relative hydrophobicity of the basic repeating unit, the alternating 1,3-linked β-d-galactopyranose and 1,4-linked 3,6-anhydro-α-l-galactopyranose or agarobiose, and its substitution by hydrophobic(methoxyl) and polar (sulfate, pyruvate) groups [208]. Agar-based thermoreversible gels have a melting point at 60–97 °C [215] and can retain their structure after freeze-drying [216]. Agar biocompartibility, biodegradability, and low toxicity has been experimentally confirmed [217].
Market situation (world market reports)	The global agar agar gum market size was estimated at USD 214.98 million in 2015 and USD 219 million in 2017.It is anticipated to grow at a CAGR of 4.9% from 2016 to 2025 [218].
Patents	Currently, about several hundreds of patents on the extraction and modification of agar and its derivatives as well as their applications exist.
	For search, use: https://patents.google.com/
	Selected examples:
	US3335127A. Fractionation of mixtures of agarose and agaropectin
	US2439964A. Extraction and preparation of agar
	US784349A. Process of manufacturing limpid solutions of agar-agar and product of same
	US3094517A.Process for treating a polysaccharide of seaweeds of the gigartinaceae and solieriaceae families
	US4780534A. Process for producing agar-agar from an algae extraction juice
	US20050267296A1.Cost-effective process for preparing agarose from *Gracilaria* spp.
	US3956273A. Modified agarose and agar and method of making same
	US3423396A. Method of making agarose
	US3281409A. Method for the separation of agaropectin from agarose
	US9045566B2. Method for the manufacture of agarose gels
	US3527712A. Dried agarose gel, method of preparation thereof, and production of aqueous agarose gel
	US3860573A. Method for cross-linking agarose or agar
	CN101891835A. Method for separating and preparing agarose from agar by using polyethylene glycol precipitation method
	US6322814B1. Manufacture of and uses for low molecular weight agars and agaroids
	GB1352613A. Stabilized agar product and method for its stabilization

Nowadays, both agar and agarose represent marine biomaterials with a high potential of their application in biomedicine and tissue engineering [213,214,217]. According to the modern view, “*agarose is particularly used as a temporary scaffold for bony cells and growth factors in the field of tissue engineering, as a biocompatible substrate enriched with osteoconductive particles for bone grafting/augmentation procedures, and as a bone spacer in guided tissue regeneration*“ [219]. Self-gelling properties and adjustable mechanical stability [220,221] of agarose gels are crucial for their use. For example, non-toxic [222] and biodegradable agarose gels have been effectively used in implantation surgery [219], wound healing, cartilage [223], cardiac, bone and nervous system [224], and regeneration as well as skin tissue engineering [225,226]. These directions are based on tunable features of agarose, which can result in adjustable characteristics similar to native tissues [225]. In addition, applications of this biomaterial for targeted drug delivery have been recently discussed in the review entitled “*Agarose-based biomaterials for advanced drug delivery*” [227]. Finally, agarose gels can be used in 3D bioprinting [228].

## 3. Marine Structural Proteins

According to a modern definition, “a structural protein is a protein that possesses a characteristic amino acid sequence or motif that repeats and forms a skeleton or contributes to the mechanical properties of a living organism, cell, or material” [229]. Typical examples of such proteins include actin, tubulin, collagen, elastin, sericin, fibroin, byssus, spongin, conchiolon, resilin, gorgonin, and keratin (see for overview [4,230]). A few selected structural proteins of marine origin are discussed below.

### 3.1. Spongin

Despite the fact that spongin (Table 7) has been studied by scientists since 1705, its true nature remains unknown and this biological material itself is attributed to one of the last mysteries of water-insoluble structural proteins that arose more than 800 million years ago, at the dawn of multicellular organisms [231,232]. The identification of spongin requires an extraordinary approach and is a challenging task that diverse research groups have failed to solve during 315 years of investigations. The low solubility of natural spongin in acids as well as after enzymatic treatments mentioned earlier [232] is a critical factor limiting its clear identification as collagen, or keratin, or a glycosylated form of one of them.

**Table 7:** Biomaterial passport: spongin.

Scientific Name	Spongin
Chemical structure	Spongin is a collagen derivative protein which can be referred to halogenated scleroproteins or neurokeratin-like proteins [231,232]. However, halogens (I, Br), detected within spongin structure, do not occur in collagens or keratins [232]. The biochemistry of spongin as well as its molecular weight remains to be unknown.
Physicochemical properties	Spongin is not soluble neither by proteases (collagenase, pepsin, trypsin, amylase, lysozyme), nor by aggressive reagents, i.e., HCl, sulfuric acid, hydrogen peroxide [233,234,235]. Treatment with alkalis dissolves spongin resulting in hydrolysates of amino acids. In the natural habitat of sponges, spongin can be destroyed by bacteria and fungi [235]. Its thermostability is species dependent and ranges between 150 °C and 360 °C [236]. Owing to spongin, the scaffolds of bath sponge *Spongia officinalis* are characterized by unique material properties, such as the ability to hold water, toughness, compressibility and resiliency [232]. Heating of spongin scaffolds up to 1200 °C under exclusion of oxygen leads to obtaining of turbostratic graphite [86].
Spongin extraction/Physical form after extraction	Spongin skeletons can be purified using 3M HCl as was shown for *Hippospongia communis* [237].
Biomaterials properties (biocompatibility, biodegradability, toxicity, immune responses)	Spongin was reported to be biocompatible, biodegradable, non-toxic and of low immunogenicity [4,232,238,239].
Market situation (world market reports)	According to Technavio report, global commercial sponge market is predicted to reach USD 3.18 billion during 2020–2024 [240]. In addition, sponges can be cultivated and such sponge farms already exist in Japan, France, Greece, the Philippines, Micronesia, Australia, New Zealand, and East Africa [232].
Patents	Currently, about several hundreds of patents on sponge cultivation, sponge scaffolds extraction, their treatments, and applications exist.
	For search, use: https://patents.google.com/
	Selected examples:
	WO2015151030A1. Method to obtain collagen/gelatin from marine sponges
	WO2006089660A2. Method for cleaning marine collagen and the treatment thereof to form porous sponges
	US20030032601A1. Method for isolating sponge collagen and producing nanoparticulate collagen, and the use thereof
	US20080261876A1. Method for purifying marine collagen and the processing thereof into porous sponges
	US20100260823A1. Preparation with marine collagen for protease inhibition
	JPH07100B2. Method of drying collagen sponge
	DE10010113A. Native sponge collagen, process for its isolation and its use, as well as native nanoparticulate sponge collagen, process for its preparation and its use

**Figure 3:**
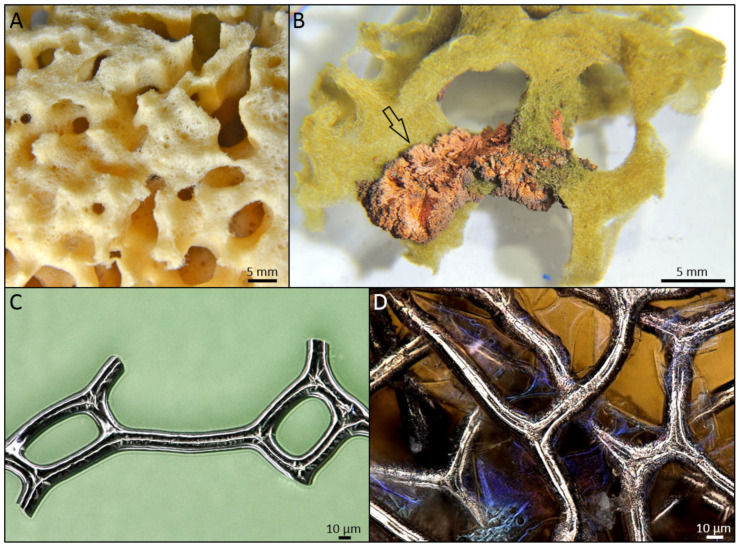
Spongin of the bath sponges origin (**A**) has been recently recognized as unique marine biomaterial for development of metal oxide-based composites (**B**, arrow) and the source for creation of mechanically stable 3D turbostratic graphite (**C**), which can be further functionalized with selected metals (**D**). For details, see [86].

Spongin represents the biopolymers with high resistance to chemically harsh and thermally extreme conditions and is one of the main players as specialized templates for extreme biomimetics (Figure 3). Nowadays, it is very important to design a bridge between extreme biomimetics and bioinspired materials science where the basic principle is to exploit chemically and thermally stable, renewable biopolymers for the development of the next generation of biologically inspired composite materials never reported, or even suggested before, with sizes and properties which will allow their application in the extremes of modern industry including a large scale level. Recent studies have revealed that especially such renewable structural biopolymers as aminopolysaccharide chitin and proteinaceous spongin can be used as thermostable biopolymeric scaffolds with 3D architecture for the nucleation and growth of a wide range of novel nanoorganized SiO_2_-, GeO_2_- Fe_2_O_3_-, ZnO-, ZrO_2_-, TiO_2_, MnO_2_, and multiphased TiO_2_/ZrO_2_-based composites (see, for an overview, [2,3,79,86]).

In particular, using an extreme biomimetic approach, the spongin scaffold of *Hippospongia communis* was coated with TiO_2_ and such new biocomposite could efficiently remove C.I. Basic Blue 9 via adsorption and photocatalysis [237]. Secondly, the application of *H. communis* scaffold as a template for hydrothermal synthesis of hematite (α-Fe_2_O_3_) resulted in the generation of a composite consisting of spongin and hematite, which was shown to enhance the electrochemical properties of the capacitor electrode [241]. Thirdly, the same template was reported to undergo extreme biomimetic treatment yielding a novel MnO_2_-spongin composite that can be employed for the development of 3D metal oxide layered biocomposites functioning as electrodes [242]. Furthermore, due to their structure composed of 3D fibrous network and to spongin perfect sorption properties, spongin scaffolds serve as excellent matrices for enzyme immobilization. Indeed, *H. communis* were studied as a template for the immobilization of *Candida antarctica* lipase B (CALB). Astonishingly, such a biocatalytic system proved to be efficient even after 20 days of storage at 4 °C: immobilized lipase catalyzed the conversion of triglycerides to glycerol and fatty acid methyl esters that is very promising for bio-fuel industry and further research focused on spongin matrix enzyme immobilization [239]. Indeed, a follow-up study using *H. communis* scaffold showed a successful immobilization of laccase from *Trametes versicolor* mushroom, which efficiently catalyzed degradation of bisphenols, toxic compounds used in polycarbonates manufacturing [243]. The removal of contaminants, i.e., phenol, chlorophenol, fluorophenol, bisphenol A was also shown in the study that exploited the properties of another biocomposite composed of *H. communis* spongin and iron phthalocyanine [244]. In addition, *H. communis* was used to construct 3D carbonized spongin-Cu/Cu_2_O scaffold that was reported to catalyze the conversion of a toxic compound, 4-nitrophenol to 4-aminophenol [86]. Recently, spongin-based scaffolds isolated from *Haliclona* sp. marine demosponge have been successfully used for preconcentration and extraction of such substances as fenitrotion [245] and ketamine [245].

### 3.2. Collagens

The most important features of marine collagens are described in Table 8.

**Table 8:** Biomaterial passport: collagens.

Scientific Name	Collagens
Chemical structure, MW	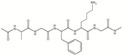
	Collagens belong to a superfamily of extracellular matrix structural proteins that are formed by a triple helix of three protein chains wrapped around each other [246,247]. Marine collagens resemble those of mammals, but their amino acid composition was shown to be much more diverse [230,231,248,249,250] MW of marine collagens, i.e., cod is about 300 kDa [251].
Physicochemical properties	Marine fish collagens are characterized by a high solubility in water upon heating, which was reported to be higher for warm-water fish species [230,252]. Incubation of collagen with thrombin results in the hydrolysis of peptide bonds and the formation of scaffolds in the form of hydrogel with a range of elasticity, transparency, and density parameters [251]. Upon heat denaturation collagen from fish, i.e., shark undergoes hydrolysis yielding gelatin [253].
Collagen extraction/Physical form after extraction	Marine collagens, predominantly type I collagen, can be isolated from marine invertebrates (sponges, jellyfish, cephalopods, echinoderms) and marine vertebrates (fish) [176,254,255]. The raw materials for fish collagen isolation include skin, scale, fins, backbone, swimbladder, wing muscles of skate, shark placoid-scale dentine [230,254]. Marine collagen is extracted via (i) decellularization using physical methods involving freezing and disruption of cells; (ii) chemical methods based on variable reagents, i.e., acids, alkalis, chelating agents, detergents, solutions of high osmolarity; (iii) enzymatic treatments. Usually, these methods are combined [230,255]. From jellyfish, it is extracted from mesoglea via solubilization in acetic acid solution [256]. The protocols for collagen extraction from sponges were reported [255,257,258]. Upon extraction, collagen or its composites have the physical form of sheets, flakes, powder, gel, particles, fibers, film, etc. [259].
Biomaterials properties (biocompatibility, biodegradability, toxicity, immune responses)	Marine collagens were shown to be biocompatible, biodegradable, non-toxic, and of weak antigenicity [255,260,261,262,263,264]. The mechanical properties of marine fish collagens can be improved by ultraviolet irradiation, gamma irradiation, dehydrothermal treatment, chemical treatment including glutaraldehyde, carbodiimide,1-ethyl-3-(3-dimethyl-aminopropyl)-carbodiimide [252,262,265] as well as incorporation of other biopolymers such chitosan, alginate, and pectin [266,267]. Unique collagen mechanical properties were reported for *Chondrosia reniformis* demosponge. It allows the species to creep and withstand compression [231].
Market situation (world market reports)	The global market for marine collagen has been steadily growing over the last years. While in 2018 it was estimated to be worth of USD 620.3 million, it is predicted to reach USD 897.5 by 2023 [268]. Primarily, marine collagen market is predicted to grow in China, India, and Brazil [268].
Patents	Currently, about several thousands of patents on marine collagen extraction, purification, modification, removing odor, improving mechanical properties and applications exist.
	For search, use: https://patents.google.com/
	Selected examples:
	US20060135752A1. Method of obtaining biologically active collagen from skins of the salmonidae fish
	DE102005041414A1. Glass sponge collagen obtained by gradually corroding glass sponge basal spicule in alkaline solution; dialyzing the obtained extract and subsequently lyophilizing, useful for the production of e.g., biological material and bullets
	DE102013014417A1. Sponge collagen comprehensive preparations with defined in vivo release profile especially in the colon, their production and use
	TWI487711B. A extraction method of collagen from tuna and product thereof
	KR101640801B1. Collagen extraction from aquatic animals
	WO2015012682A2. A method for extracting collagen from aquatic animals, collagen and products containing it
	JP4236850B2. Method for producing fish-derived collagen peptide, and food and drink and cosmetics containing fish-derived collagen peptide obtained by the method
	EP0592586B1. Use of unpigmented fish skin, particularly from flat fish, as a novel industrial source of collagen, extraction method, and collagen and biomaterial thereby obtained
	CN1582771B. Production of collagen peptide from fish skins
	US9591853B2. Jellyfish-derived polymer
	EP2889305A1. Method for fractionally extracting mucin and collagen
	WO2009090655A2. Colloidal collagen burn wound dressing produced from jellyfish
	US5714582A. Bioscience Consultants Invertebrate type V telopeptide collagen, methods of making, and use thereof
	JP2007504100A. Medical and insurance use of pufferfish type I collagen extract and method for producing the extract
	KR100381741B1. Collagen product containing collagen of marine origin with a low odor and with improved mechanical properties, and its use in the form of cosmetic or pharmaceutical compositions or products

#### 3.2.1. Marine Invertebrates Collagen

Recently, the biocompatibility and cell responses to marine invertebrate collagens have been reported in different studies. For example, employing murine fibroblast cells, biocompatibility of cryogels composed of jellyfish collagen, chitosan, and fucoidan was demonstrated [159]. Poriferan collagenous scaffolds, on the other hand, represent natural 3D scaffolds with a great potential for tissue engineering [263]. In particular, in vitro experiments using primary murine osteoblasts demonstrated a good cell attachment and proliferation when cultured on sponge collagenous scaffolds [269]. A series of experiments revealed a positive effect of sponge collagen hydrolysates on damaged or photoaged skin [270]. In addition, a recent in vivo study using rats demonstrated biocompatibility and the ability to support bone formation of biocomposites generated from collagen, isolated from A. fulva, and biosilicate [271]. Finally, powdered collagenous sponge scaffold loaded with L-cysteine hydrochlorid proved to cause a positive effect on wound healing [272].

Intriguingly, sponge collagen served as a template in several scientific projects aiming at the generation of bioinspired silica layered composite biomaterials [261] that resemble naturally occurring poriferan biocomposites [273,274]. For example, in laboratory conditions, collagen of different origin, i.e., isolated from *Chondrosia reniformis* marine demosponge, underwent in vitro silicification resembling the growth of siliceous spicules in glass sponges, which is promising for the generation of new collagen-silica hybrid materials on industrial scale [260,275]. Moreover, a specific amino acid motif, Gly-3Hyp-4Hyp, was discovered within the glass rope sponge Hyalonema sieboldi collagen, which presumably is predisposed for silica precipitation [276]. Thus, the modification of collagen amino acid sequence might significantly improve the construction of siliceous spicules layered biocomposites.

#### 3.2.2. Marine Vertebrates Collagen

Both fishery and mariculture of selected fish species represent important sources of collagens (see for overview [254]). Marine fish collagen-based biomaterials (i.e., collagen gels, scaffolds, sponges, films, membranes, and composites) have a wide range of applications including drug delivery, wound healing, wound dressing, tissue engineering, i.e., bone, cartilage, dental, vascular and skin tissues, and therapeutics against skin aging, diabetes, and obesity [256,262,277,278].

The use of marine wastes including by-products of industrial plants, such as fish skin, scales and fins, as a source of fish collagen helps to fight environmental pollution and serves as a strategy to valorize marine resources [254,279]. Intriguingly, it is possible to isolate fish collagen from skin of marine Eel fish [280], codfish [281,282,283], European hake [284], smooth wolf herring [267], blue shark [285,286], small-spotted catshark [253], salmon [266,283], ocellate puffer fish, seaweed pipefish, brownstripe red snapper, brownbanded bamboo shark, carp, largefin longbarbel catfish, Japanese sea-bass, bigeye snapper, surf smelt, brown backed toadfish, Nile perch, skate, blacktip shark [255,256], bones of European hake [284], carp, Japanese sea-bass, skipjack, ayu, yellow sea bream, horse mackerel, Baltic cod [255], swim bladder of Atlantic cod [287], cartilages of brownbanded bamboo shark, blacktip shark, scales of carp, tilapia, spotted golden goatfish, grey mullet, rohu, and catla [255,256].

The application of fish collagen as biomaterial in biomedicine including tissue engineering has been thoroughly studied. Indeed, using cell culture assays, it was shown that 3D printed fish collagen/alginate scaffolds proved to be biocompatible with human MSCs [280]:

1. 3D printed scaffolds consisting of fish collagen/alginate and phlorotannin (as a bioactive component) displayed good biocompatibility and stimulated osteogenic differentiation of osteoblast-like MG63 cells [288];
2. 3D printed fish collagen/alginate hydrogels containing murine fibroblasts were of good biocompatible characteristics [285];
3. fish collagen was reported to be biocompatible with human fibroblasts [282];
4. 3D printed scaffolds composed of fish collagen and calcium phosphates derived from two sharks, blue shark and shortfin mako shark, were biocompatible with osteoblast-like Saos-2 cells [286];
5. composite scaffolds from fish collagen and chitosan promoted osteogenic and chondrogenic differentiation of rat MSCs [266];
6. fish collagen composites cross-linked by genipin under CO_2_ atmosphere were biocompatible with murine chondrocytes [253].

Fish collagen is also employed in dentistry, usually as membranes and bone graft materials [257,269]. Furthermore, this structural protein is used for controlled drug release including antimicrobial agents such as tetracycline [270]. In another research, a potential of anticancer drug(s) loaded 3D printed patches from fish gelatin for anticancer treatment was demonstrated [271]. Due to its excellent absorption properties and the ability to resorb up to 56 days, fish collagen can be used to control wound blood bleeding [272]. In addition, it has a high potential for cosmetic applications: fish collagen demonstrated a moisturizing effect without irritating skin [263].

### 3.3. Gelatin

Gelatin (Table 9) can serve as cell carrier to repair tissue defects, i.e., gelatin extracted from marine snail *Rapana venosa* was reported as a biocompatible template for the growth of human keratinocytes [289]. Hence, this marine biomaterial can be used in tissue engineering, often in combination with other materials such as chitosan and silk fibroin [42]. Indeed, chitosan/gelatin and silk fibroin/gelatin composites were employed in hepatocytes research and can be applied to generate 3D hepatic microenvironments, which would shed more light on hepatic cell functions [290]. Importantly, marine gelatin can be used in the inhibition of angiotensin-converting enzyme in order to lower blood pressure and reduce the risks of myocardial infarction, congestive heart failure, stroke, and arteriosclerosis [291]. Amino acid sequences of peptides inhibiting angiotensin-converting enzyme were detected in the studies on gelatin extracts of Alaska pollack [291] and can be further applied to prevent hypertension.

**Table 9:** Biomaterial passport: gelatin.

Scientific Name	Gelatin
Chemical structure, MW	(C102H151O39N31), the amino acid sequence of gelatin depends on its source and is similar to that of collagen, comprising of repeating sequences of Gly-X-Y triplets, where X and Y are represented by mostly proline and hydroxyproline, respectively. The average MW is in the range of 40 to 700 kDa [292,293].
Physicochemical properties	Gelatin properties vary in a broad spectrum, depending on the material used, pretreatment method, extraction process parameters and its intensity. Reported pH values span from 2.98 to 4.38; isoelectric point for acid-processed gelatins is in the pH range of 6.0–9.5, while for alkali-processed gelatins it falls between the pH of 4.8 and 5.2, moisture content is in the range of 9–14% [292,293].
Fish gelatin extraction/Physical form after extraction	Gelatin extraction was reported from various marine species, i.e., fish species [256,294], sponges [295], jellyfish [296] and other marine organisms such as squids [297] and snails [298]. The processing with alkaline or acidic media in elevated temperatures yields gelatin in the form of granulates or powders [292,293].
Biomaterials properties (biocompatibility, biodegradability, toxicity, immune responses)	Fish gelatin is considered to be biodegradable, non-immunogenic and biocompatible [299,300,301]. It does not display toxicity or carcinogenicity and has very poor mechanical properties, dependent on the source type (cold/warm fish) or experimental conditions; e.g., tensile strength varies from 36.8 MPa for the cold-water pollock derived gelatin to 95.5 MPa for the catfish [302].
Market situation (world market reports)	Production of fish gelatin is still quite small, contributing only to ca. 1% of the global gelatin market [292,293].
Patents	Currently, about several hundreds of patents on utilization of fish gelatin in food and pharmaceutical industry as components of packaging systems or drug delivery, medicine and cosmetics are available.
	For search, use: https://patents.google.com/
	Selected examples:
	US20030022832A1. Method for the production of gelatin of marine origin and product thus obtained
	JP4738005B2. Fish skin pretreatment method
	JP6265350B2. Extraction method of collagen and gelatin
	TWI487711B. A extraction method of collagen from tuna and product thereof
	US6368656B1. Process for the preparation of fish gelatin
	WO2017216780A1. Gelatin polymer derived from natural sources of cold-adapted marine species and uses thereof
	WO2012160575A2. Method of producing gelatin from fish
	US5093474A. Process for the production of gelatin from fish skins
	US20050124034A1. Method for producing fish gelatin peptide
	CN104605006A. Freeze-drying method for swim bladder
	WO2019022623A1. Process for producing gelatin from fish skin by optimisation of the extraction conditions
	US2048728A. Process for making a clear fish glue or fish gelatin solution
	CN102702984A. Process for industrially producing fishskin gelatin
	GB2377708A. Improved alkaline process for preparing type B fish gelatin
	US5484888A. Gelatin production

Moreover, due to its gel-forming properties, marine gelatin is also applied in food industry as a stabilizer, texturizer, thickener and foaming agent in yoghurt, ice-cream, jam, cream cheese, marshmallows, etc. [303,304]. Presumably, due to the lower content of proline and hydroxyproline in comparison to beef- and pork-derived gelatins [294,305], marine gelatins form “weaker gels” [306,307]. Notably, gelatin inhibits peroxidation preventing food from deterioration and functions as an outer protective film against dehydration, oxygen, and light [304]. In addition, isinglass, a high-grade gelatin derived from fish swim bladders that can induce aggregation of yeast and other insoluble particles, can be widely applied as a commercial clarifier in beverages, i.e., wine, beer, cider [303]. Though marine gelatin may trigger allergy, i.e., 0%–8% incidence depending on local food habits and fish consumption is reported [304]. Finally, marine gelatin is widely used in capsule industry. Usually, it is applied for the encapsulation of temperature-sensitive vitamins and other nutrients [308].

### 3.4. Keratin

Keratin (Table 10) is a fibrous protein of a high importance in the animal kingdom. Keratin presence in horn, hoofs, hair, beaks, shells, toenails, claws, fingernails, and feathers renders it the most abundant structural protein [309]. In such marine mammals as whales, keratin is to be found as the main structural component of baleen (see, for details, [230]). In general, keratin is present in two forms, characteristic for the type of tissue it is present in: α-keratin, found in soft tissues, e.g., wool, hair or skin, and β-keratin dominating in feathers, nails, fish scales, and other hard tissues. Structurally, both keratin types show a filament-type matrix structure. However, α-keratin filaments, denoted as intermediate filaments (IF), are two times greater in diameter (7–10 nm) compared to β-keratin filaments diameter of 3–4 nm. From the mechanical point of view, keratins have high strength and stiffness; the properties typical for the tissues keratin is a component of [310].

**Table 10:** Biomaterial passport: keratin.

Scientific Name	Keratin
Chemical structure, MW	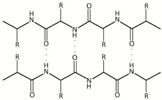
	Fibrous structural protein of a molecular weight ca. 66.6 kDa [311]
Physicochemical properties	Keratin is a stable protein, insoluble in polar and nonpolar solvents [311].
Keratin extraction/Physical form after extraction	Depending on the source, keratin extraction is quite a demanding process and its parameters influence the scope of application of the extracted keratin, available as a powder or liquid [312,313].
Biomaterials properties (biocompatibility, biodegradability, toxicity, immune responses)	Keratin is biodegradable [314], biocompatible and non-toxic [315]. Reported properties of keratin differ depending on its source. Young’s modulus ranges from 10 MPa in stratum corneum to about 2.5 GPa in feathers; tensile strength varies from 2 MPa in stratum corneum to 530 MPa in dry hagfish slime threads [310]. Reported stiffness of keratin is up to 20 GPa [316]; however, it strongly depends on the level of hydration [317]: for hagfish slime threads the initial stiffness reaches 3.6 GPa in dry state and drops to 6 MPa in wet state [310].
Market situation (world market reports)	There are no open access reports on the marine keratin market situation.
Patents	Currently, about several hundreds of patents on utilization of keratin in cosmetics, hair care products, adhesives, wound dressing or as components of antibacterial and anti-inflammatory products are available.
	For search, use: https://patents.google.com/
	Selected examples:
	US7148327B2. Production of soluble keratin derivaties
	CN1535280A. Production of soluble keratin derivatives
	WO2019116357A1. Method for extracting keratin
	US8575313B2. Process for extracting keratin
	US20140228257A1. Method for Sea Floor Drilling Using Hagfish Slime as Drilling Fluid Additive
	US7049405B2. α-helical protein based materials and methods for making same
	CN106999546A. Keratin nano material and preparation method thereof
	WO2007095151A2. Nerve regeneration employing keratin biomaterials
	US20100197021A1. Keratin biomaterials for cell culture and methods of use
	US6110487A. Method of making porous keratin scaffolds and products of same
	US8920827B2. Keratin bioceramic compositions

Though keratins seem to be constituents of static matrices (i.e., baleen) [310], there are exceptions to this rule such as hagfish. Hagfish (Myxinidae) are deep water inhabiting living fossils the body of which has an eel shape with no scales present. The remarkable feature of hagfishes is their ability to, when provoked or threatened, produce and excrete large amount of slime consisting of keratin IFs. The filaments act as threads binding mucin, a protein capable of forming gels [318]. When shot out of the slime gland followed by the contact with seawater, the slime becomes extremely dilute and is capable of effectively covering or choking the hagfish predator almost instantly. Detailed mechanical analysis of hagfish threads reveals their remarkable mechanical properties, different in dry state when compared to their wet state. In particular, dry hagfish threads show high initial stiffness of 3.6 GPa and a high tensile stress of 530 MPa while wet threads exhibit stiffness of 6 MPa and tensile strength of 180 MPa [317]. The outstanding mechanical properties of dry threads combined with the ease of their synthesis have been the reason for considering hagfish slime as a substrate for engineering fibers acting as a reinforcement for various modern composite materials [319].

Due to its poor solubility and tedious extraction methods, keratin has so far found limited applications; nonetheless, attempts were made to expand its usefulness. Initial studies on potential applications were focused on cells and their behavior on keratin containing films [320] and further extended on the potential of these films to act as active molecule carriers [321] or focused on altering their mechanical and antibacterial properties [322]. Keratin films have also been proposed for ocular surface reconstruction due to their good corneal biocompatibility and transparency [323].

### 3.5. Conchiolin and Conchixes of Molluscan Origin

To solve the problem of conchiolin insolubility, numerous hydrolysis protocols yielding soluble peptides of better functionality and applicability were developed: hydrolyzed conchiolin protein of pearl shell origin is a common cosmetic ingredient of hair and skin conditioning agents [324] or cleansing solutions [325]. More detailed studies on the properties and possible future applications of molluscan matrix protein extracts have been performed by Latire and co-workers [326]. In the first study, the authors analyzed shell extracts (acid soluble (AS), acid insoluble (AI) and water soluble (WS)) from the marine bivalve *Pecten maximus* [326]. AS did increase human fibroblast metabolic activity following 24 h of incubation. Likewise, the extracts obtained from mussel *Mytilus edulis* (AS and WS) and oyster *Crassostrea gigas* (AS) led to an increase in primary human skin fibroblast metabolic activity and cell proliferation [327]. Such data indicate the potential applications of these matrix proteins or their extracts in medicine, especially in wound healing or the treatment of various skin conditions. Indeed, in vivo study employing rats with dorsal skin wounds [328] demonstrated a progressive wound reduction after the ointment containing powdered shells of *Megalobulimus lopesi* was applied. This effect was attributed to calcium, which, after being administered to the wound tends to enhance the healing process [329,330], though the authors do not rule out the possibility of so-called *conchix* proteins to be involved in the facilitation of the healing process. *Conchix*, a term representing the shell organic matrix, has been recently proposed by Ehrlich and co-workers [331] to underline the importance of this organic piece of mollusc shell architecture. A brief summary on conchiolin properties is provided by Table 11.

**Table 11:** Biomaterial passport: conchiolin.

Scientific Name	Conchiolin
Chemical structure, MW	Conchiolin is reported to be an aggregate of proteins including a significant portion of polysaccharide component [332]. When isolated from mollusk tissue and separated by PAGE, it gives three main protein bands with molecular weight of 37.8, 23.2, and 19.6 kDa. The amino acid analysis of the isolated material shows the presence of high content of glycine and alanine (30–60%) and a large number of hydrophobic residues [332].
Physicochemical properties	Insoluble in water and acid [333].
Conchiolin extraction/Physical form after extraction	Conchiolin can be extracted form ground mollusk shells by subsequent washing with EDTA solution, basic Tris buffer and water followed by the extraction with SDS solution at increased temperature to yield conchiolin as a powder [332].
Biomaterials properties (biocompatibility, biodegradability, toxicity, immune responses)	Due to their biocompatibility, marine collagens can be applied in biomedicine, regenerative medicine, wound healing, cartilage and hard tissue engineering. Domains typical for collagen have been detected as main structural segments in other structural marine proteins including conchiolin [4]. This may suggest that conchiolin may exhibit properties similar to collagen that is highly biocompatible and applicable as a biomaterial. Conchiolin is a calcium binding protein which facilitates calcification during shell formation thus exhibiting a potential to be applied in bone engineering [334,335].
Market situation (world market reports)	Today’s market exhibits fast increase in the demand on medical devices supporting the regeneration of bone fractures and defects [336]. Due to its calcium binding properties [334], conchiolin exhibits the potential to be applied as a component of bone regeneration scaffolds.
Patents	Currently, several patents on conchiolin extraction, modification and application exist.
	For search, use: https://patents.google.com/
	Selected examples:
	US20110274792A1. Method for producing powder for supplementary food and supplementary food
	US5702728A. Clam extract preparation, the method of preparation and use thereof
	WO2010005243A2. Method for producing an extract containing water-soluble conchiolin derived from shells
	EP3228625A4. Preparation method for conchiolin, and water-soluble conchiolin and acid-soluble conchiolin prepared by using method
	FR2827478B1. Process for the preparation of a nacre-based powder, isolated protein from said powder and their uses in bone surgery and in various osteoarticular pathologies

The molecular complexity of the conchix hinders its applicability, especially in highly regulated industries, e.g., pharmacy. Dealing with this issue requires an intense focus on the isolation of active matrix components [337].

The optimistic conclusions resulting from the above-mentioned studies are challenged by the vast number of conchix active components which are extremely difficult to get rid of in order to isolate a single substance exhibiting biomedical or cosmetic potential. This fact is reflected in a small variety of products utilizing shell proteins as active components.

## 4. Marine Biominerals

**Figure 4:**
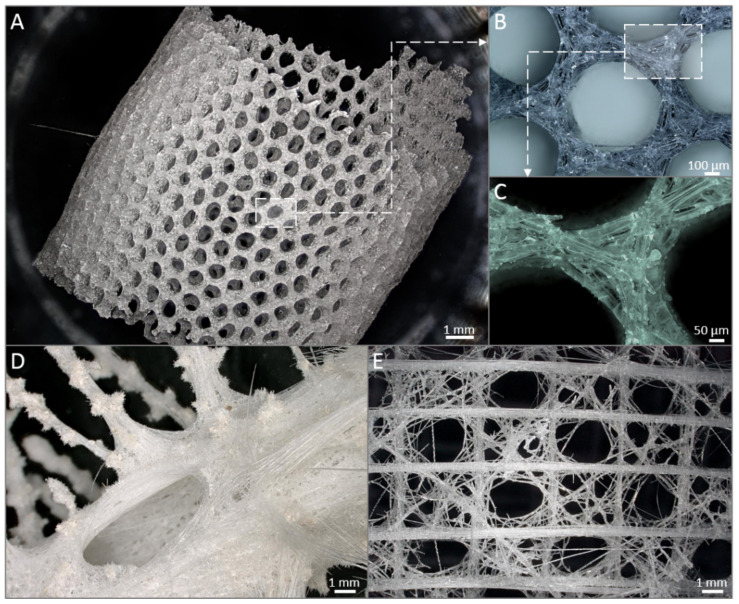
Digital microscopy images: Despite protection and the lack of industrial harvesting, glass sponges, thanks to the complex architecture of their biosilica-based skeletons (**A**–**C**)—*Aphrocallistes* sp., (**D**)–*Waltheria* sp.; (**E**)—*Euplectella* sp.) represent a unique source for creating 3D models for potential biomimetic functional materials.

Biominerals have been recognized as the main players in skeletogenesis of diverse organisms including those inhabiting seas. Being the products of vital activity of cells and specialized tissues, they are formed as the result of the interaction of organic matrices with various mineral phases regulated at the molecular and genetic level. Biosilica, calcium carbonates, and phosphates (mostly in marine vertebrates) represent the dominant mineral phases in a broad diversity of biocomposite-based skeletal constructs. In contrast to Ca-based biominerals discussed below, we paid no attention to highly sophisticated biosilica- based constructs of sponges origin [276,338,339,340,341,342] which represent diverse biomimetic models (Figure 4); however, they are not industrially harvested, or cultivated being mostly protected.

### 4.1. Corals

Corals (class Anthozoa) are marine invertebrates offering great opportunities for biomedical applications. “Coralline biomaterials” [343] (see also Table 12) have been well recognized in biomaterials science community (see, for an overview, [4,42,166]).

**Table 12:** Biomaterial passport: Coral biominerals.

Scientific Name	Coral Biominerals
Chemical structure, MW	Coral skeletons are composed mainly from CaCO_3_. MW: 100.1 g/mol [344].
Physicochemical properties	Coral material is quite stable. It preserves highly organized porous structure after hydrothermal treatment and even sintering at 1250 °C [345]. Hydrothermal treatment of as sea received coral samples results in the transformation of crystalline aragonite (CaCO_3_) to hydroxyapatite [345].
Coral extraction/Physical form after extraction	Coral derived materials include coral hydroxyapatite and aragonite, natural coral fragments, coral granules and coral powders [346].
Biomaterials properties (biocompatibility, biodegradability, toxicity, immune responses)	Coral-derived material is biocompatible, structurally similar to human bone, with Young’s modulus of 0.580 to 9.032 GN m^−2^ (reported for octocorals) [347], non-toxic, biodegradable and of low immunogenicity [4,348]. Mechanical properties of octocorals were shown to depend on environment, i.e., the stiffest skeletons belong to the inhabitants of deeper environments (with pressure >80 atmospheres) while the least stiff skeletons are found in the colonies from shallow environments with moderate waves [347].
Market situation (world market reports)	Materials to reconstruct bone defects are in high demand. In 2021, global markets for orthopedic and dental bone graft products is predicted to reach USD 3.4 billion and USD 1.0 billion, respectively [349]. Bone allografts can be obtained from corals cultured in aquarium systems and enriched with silica and strontium increasing coral osteoconductive properties, which was patented in the U.S.A. and Israel [349].
Patents	Currently, about several hundreds of patents on coral cultivation, hydrothermal treatment of coral material yielding hydroxyapatite, modification of coral material and its applications exist.
	For search, use: https://patents.google.com/
	Selected examples:
	WO2009066463A1. Method of producing coral powder
	CN-107951818-A. Reparation toothpaste containing coral powder and hydroxyapatite component and preparation method thereof
	WO2010078879A2. Cosmetic use of a coral powder
	US8936638B2. Coral bone graft substitute
	EP2618858B1. Coral bone graft substitute
	WO2009066283A2. Calcium-mediated effects of coral and methods of use thereof
	KR100536500B1. Mass propagation methods of Korean Corals
	JP2008141989A. Method for propagating coral
	CN101702998B. Propagation method for coral grass seedling tissue culture
	WO2009066283A3. Calcium-mediated effects of coral and methods of use thereof
	EP0952114B1. Weathered hermatypic coral material
	DE20311110U1. Biological dental implant consists of coral
	US20060147656A1. Simulated coral rock and method of manufacture
	RU2472516C1. Biomaterial for bone defect replacement
	US7608283B2. Coral purification method and coral thus obtained
	WO2002040398A1. Processes for treating coral and coating an object

Coral skeletons are often used as the sources for inspiration to create artificial 3D constructs “with a 3D bioprinting platform which mimics morphological features of living coral tissue and the underlying skeleton with micron resolution, including their mechanical properties” [350]. The use of bioceramics of coral origin represents an attractive alternative to metal-based constructs [351] for implantology and tissue engineering [352]. Furthermore, the coral structure can undergo chemical conversions yielding calcium phosphate particles, which could be used in tissue engineering and as drug carriers: the conversion of Tubipora musica coral at 400 °C and 800 °C resulted in plate-like calcium phosphate nanoparticles (mostly Monetite) and spherical shaped calcium phosphate nanoparticles (whitelockite and hydroxyapatite), respectively [353]. Likewise, the scleractinian corals, *Porites* spp., were converted to hydroxyapatite employing hydrothermal and mechanochemical treatments [353]. Hence, the properties of coral skeletons inspire a whole range of studies focused on coral bone graft substitutes [354] as well on osseointegration with human bones [344,352,353,355,356,357]. Indeed, in vivo study demonstrated efficient bone formation at critical size defects in sheep bone using MSCs-covered scaffolds from Acropora coral [358]. Another research demonstrated that murine preadipocytes cultured on coralline skeletal material obtained from Porites lutea corals differentiated into osteoblasts [359]. In addition, positive effect has been obtained with respect to activity of human osteoblast-like MG-63 cells growing on the scaffolds isolated from the coral *Goniopora* sp. [360]. Moreover, as recently reported by Gancz and co-workers, “the coral skeleton biomaterial may act as a strong, promotive scaffold for tissue regeneration due to its ability to reduce its rejection by inflammatory reactions such as phagocytosis” [361].

In addition to application of stony corals reported above, octocorals also possess a high biomimetic and biomedical potential [4]. Indeed, their structural architecture, the role of gorgonin-associated mineralization, and the potential of deep-sea bamboo octocoral for tissue engineering were reported [346]. The skeleton structure of black coral species, i.e., *Parantipathes larix* [27], or *Cirrhipathes* sp. [25] contains chitin that was also shown to be biocompatible and serves as a template for cell adhesion and differentiation.

Large scale production of coral-based biomaterials is limited due to the protection of coral reefs [362]. However, further investigations to use corals as model 3D porous constructs and source for bioinspiration in materials science are trending well.

### 4.2. Molluscan Shells

Though molluscan shells (Table 13) have been intensively studied primarily as the indicators of environmental transformations [363,364] and contamination [365,366]; over the years, this has changed with the focus on biomechanics [367,368,369,370], biomimetics, and materials science [26,371,372,373,374,375,376] of these biomineralized constructs.

**Table 13:** Biomaterial passport: Molluscan shell.

Scientific Name	Molluscan Shells
Chemical structure, MW	Mostly composed of CaCO_3_, MW: 100.1 g/mol [377].
Physicochemical properties	Molluscan shells are stable, exhibiting a high degree of morphological and crystallographic ordering [378] resulting in high values of the elastic modulus and bending strength (up to 82 GPa and 267 MPa, respectively) [379,380]. Importantly, the quality of the shell and its physical properties depend on environmental conditions [381]. High temperature treatment of shells leads to the conversion of CaCO_3_ to calcium oxide (CaO) [382] or it can be converted to hydroxyapatite by the hydrothermal method [383].
Molluscan shell extraction/Physical form after extraction	In general, molluscan shells are collected as aquaculture industry waste byproduct and are further processed [384]. Physical forms of shells include shell fragments and powders [384].
Biomaterials properties (biocompatibility, biodegradability, toxicity, immune responses)	Molluscan shell derived materials are considered to be biocompatible [385]. The nacre was reported to be biocompatible, biodegradable and exhibit osteogenic properties [386]. Furthermore, it showed limited cytotoxicity [387] and did not elicit immune responses [388]. The nacre exhibits outstanding mechanical properties which are species dependent (*Pincfada*: tensile strength of 140–170 MPa, Young’s modulus of 60–70 GPa; *Hydnum rufescens*: tensile strength of 180 ± 20 MPa; *Pinctada margaritifera*: tensile strength of 220 ± 60 MPa) [386].
Market situation (world market reports)	The variety of molluscan shells applications (poultry food, pet nutrition liming agents) created a market of potentially increasing demand [389]. The development of shell valorisation methods will be crucial for the market stabilization [384].
Patents	Currently, about several hundreds of patents related to various application of molluscan shells (building material component, bone graft material, decontaminants) or nacre itself (composites, cosmetic ingredients) exist.
	For search, use: https://patents.google.com/
	Selected examples:
	CN101971982A. Oyster shell powder containing hydrogen and manufacture method thereof
	CN106866807A. The preparation method of pearl protein, the pearl protein prepared by the method and its application
	WO2008017962A8. Microcapsules with improved shells
	KR101357078B1. Process for seperation of cutoffs having anti-inflamentary or osteoarthritis inhibition effects using oyster shells
	KR101771055B1. Composition comprising water-soluble pearl powder for skin whitening, anti-inflammation and anti-aging
	UDS 5968772. Pearl protein (nacrein) and process for producing the same
	US4312099A. Process for shucking a mollusk
	US8067078B1. Nacre composites, methods of synthesis, and methods of use
	US 6251438. Method of preparing active substances from nacre, products obtained which can be used in particular as medicaments
	FR2777190B1. Extraction process, identification of the active ingredients contained in the internal and external shell of sea molluscs, their use in people-based thera, diagnosis and cosmetic preparations
	FR2799125B1. Process for the preparation of a composition by extraction of nacre, comprising the complete components of the nacre, composition obtained by this process and its use in pharmacy and cosmetics.
	FR2899478A1. Process for extracting nacre molecules, compositions and use
	US8162241B2. Apparatus and method for collecting and crushing seashells on a beach
	US4939814A.Cultured mussel cleaning machine
	WO1997015398A1.Method for producing a lime product from mussel- and/or seashells

With about 18 million tons of total annual production, shelled molluscs are one of the most important components of global aquaculture industry [390], being especially important for the regions of Eastern Asia (China, South Korea and Japan), and, to a less extent, North America (the USA) and South America (Chile). Due to the risks associated with freshwater shortage, energy consumption and constantly increasing human population the development of offshore mollusc farms is of an increasing interest. In order to valorize molluscan shell wastes that constitute from 59% up to over 75% of the total organism weight depending on molluscan taxa [391], several solutions were developed.

**Figure 5:**
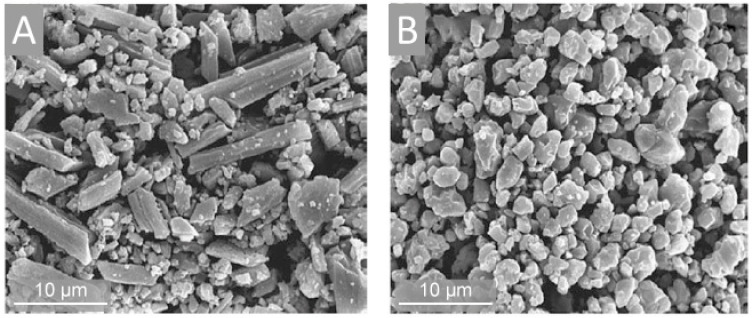
SEM micrographs showing morphology of the particles in (**A**) mussel and (**B**) quarry derived limestone. Images adapted with permission from [393].

Being composed mainly from calcium carbonate (it can reach up to 99.9% of the shell mass), shell wastes can be utilized as a mortar component [392]. The authors revealed that crushed oyster shell small particles (0.074–2 mm) were more suitable than the large ones (2–4.75 mm) as sand substitutes. A similar study conducted by [393] demonstrated that mollusc based CaCO_3_ particles are longer and of prismatic shape in contrast to the round and shorter particles of traditionally used limestone (Figure 5), thus affecting mortar setting time and its mechanical properties.

The use of shell CaCO_3_ as a source of calcium in livestock feed supplements is another alternative to utilize shell wastes. Such supplements were reported to have a positive impact on animal bones and the strength of eggshells [394]. In addition, different studies indicated that both oyster and clam shells are equally effective regarding eggshell strength and egg production rate [395,396,397,398,399]. Such benefits of shell derived CaCO_3_, shown in earlier studies, were also confirmed in recent research [400,401].

Furthermore, molluscan shells can be applied for soil neutralization and metal decontamination. Soil neutralization, typically known as liming, is performed in order to reduce acidity, improve oxygen levels, soils fertility and structure, therefore directly affecting agricultural crop yields [402]. Indeed, the use of crushed oyster shells leads to an increase in soil pH, available phosphorus and exchangeable cations, thus, positively affecting the productivity of Chinese cabbage [403]. In addition, marine shell wastes can be applied as soil decontamination agents as was reported for soils containing copper [404], lead, cadmium [405], and arsenic (V) [406].

Another option to utilize molluscan shells lies in wastewater filtration. Several studies pointed to the capabilities of razor clam and oyster shells to accumulate Zn^2+^, Pb^2+^, and Cd^2+^, though with different capacities, favoring calcite rich oyster powder for Pb^2+^ and aragonite rich razor clam for Cd^2+^ [407].

Intriguingly, molluscan shells, being among the most important biominerals known to date, challenge man-made ceramics, i.e., they are characterized by a specific internal features hierarchy, structural organization, and organic-mineral phase interactions that are formed in mild conditions unlike ceramics, which require high temperature and/or high pressure [408].

**Figure 6:**
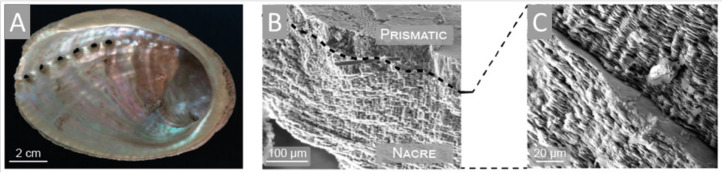
Abalone (*Haliotis* sp.) shell and its structure. (**A**) interior view of the shell and (**B**) scanning electron microscope micrograph showing the cross section the shell; (**C**) the microstructure of the nacre. Image reproduced with the permission from [412].

**Figure 7:**
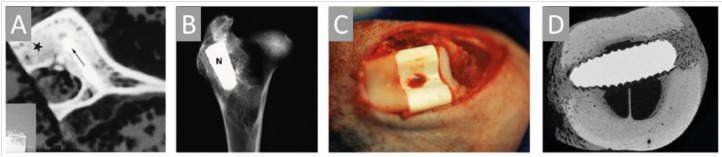
Examples of different forms of nacre used in bone graft studies. (**A**) nacre powder (denoted with a ★) injected within sheep vertebrae; adapted from [413], with permission; (**B**) nacre in the form of a cylinder (N) implanted in sheep femoral epiphysis; adapted from [388] with permission; (**C**) trochlea shaped nacre in sheep femoral trochlea; adapted from [414] with permission; (**D**) screw shaped nacre implanted in sheep metatarsus; adapted from [415] with permission.

The nacre, of the structural shell elements (Figure 6), has gained special attention. An increasing interest in the nacre, an acellular composite of calcium carbonate acting as an internal shell coating for bivalves, cephalopods and gastropods, stems from its structural similarity to bone and remarkable mechanical properties, i.e., Young’s modulus of 30–40 GPa for the nacre vs. about 20 GPa for human bones, resistance to failure of 185–200 MPa for the nacre vs. about 140 MPa for human bones [409,410]. Hence, nacre material has a great potential to be used as bone grafting material (Figure 7) [411].

**Figure 8:**
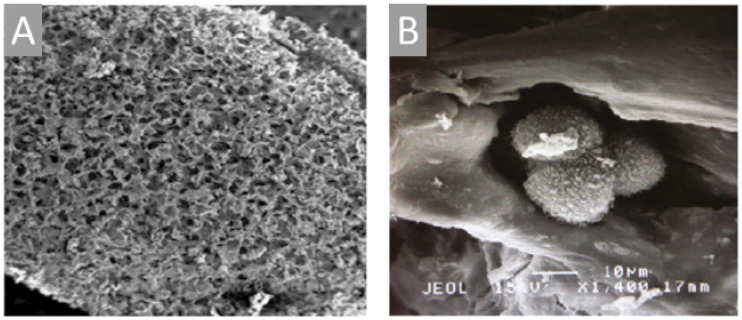
SEM micrographs presenting (**A**) porous morphology of fabricated scaffold and (**B**) cells attached to the surface of the scaffold matrix—figures adapted from [385] with permission.

Indeed, osseointegrative properties of the nacre and its potential for implantology were shown in numerous studies [411,413,414,416,417,418]. Moreover, molluscan shells can be used in bone biocomposite scaffolds [385,419,420], which are characterized by porosity favouring cell seeding and adhesion (Figure 8).

## 5. Conclusions

Biological materials of marine origin represent a special scientific niche within the global biomaterialogy with a long history of their research and applications in diverse fields of human activity.

What was recently referred to as processed marine biological waste is now considered raw material for the production of biomaterials, which differ from their synthetic analogs in biocompatibility and possess excellent biodegradability. In addition, the approach to the study of marine biocomposite structures has shifted to the point of view of modern bionics and biomimetics, when these materials are thought of as models for creating new composites, which are produced according to «drawings drawn by nature» as a result of evolutionary selection. The design of such new hybrid materials is of crucial importance for fundamental science because further progress in their research and application is impossible without understanding the mechanisms of their formation as well as their structural features at the molecular and nano-level.

The progress in marine biomaterials research is mainly attributable to its strong interdisciplinary character: the exchange of expertise in marine and structural biology, bioinspired materials chemistry, biomineralogy, biomimetics, biomechanics, and solid state physics is a key action to strengthen the scientific and practical level of this modern research field. We are strongly convinced that the scientific area described herewith will include both a high degree of novelty and challenging tasks in the future. Researchers will discover the key principles of molecular structure of marine biomaterials that will finally let them realize the dream of understanding the chemistry and materials science of diverse unique marine biocomposites spanning from atomistic detail to the macrolevel.

This concept will adopt a truly multidisciplinary and multi-scale approach to study not only the structural peculiarities of marine biomaterials, but also the mechanisms of their transformation in hybrid, functionally advanced composites, hierarchically constructed during special treatments and modifications according to human goals. A holistic understanding of the creation of a new generation of bioinspired composites and its impact on large-scale biomimetics with future input in modern technologies can only be achieved by a modern multi-facetted approach, which has not been attempted before. An undoubted factor stimulating progress in marine biomaterials is a sharp move away from synthetic plastic materials due to the serious and global threat of pollution of the world’s oceans by microplastic waste. We readily believe that biomaterials of marine origin will also be actively studied because of their extreme prospects for the so-called marine bioeconomy worldwide.
